# High Rate of Infection by Only Oncogenic Human Papillomavirus in Amerindians

**DOI:** 10.1128/mSphere.00176-18

**Published:** 2018-05-02

**Authors:** Daniela Vargas-Robles, Magda Magris, Natalia Morales, Maurits N. C. de Koning, Iveth Rodríguez, Tahidid Nieves, Filipa Godoy-Vitorino, Gloria I. Sánchez, Luis David Alcaraz, Larry J. Forney, María-Eglée Pérez, Luis García-Briceño, Leen-Jan van Doorn, María Gloria Domínguez-Bello

**Affiliations:** a Department of Biology, University of Puerto Rico, San Juan, Puerto Rico, USA; b Servicio Autónomo Centro Amazónico de Investigación y Control de Enfermedades Tropicales Simón Bolívar, MPPS, Puerto Ayacucho, Venezuela; c DDL Diagnostic Laboratory, Rijswijk, The Netherlands; d Ministerio del Poder Popular para la Salud, Caracas, Venezuela; e Department of Natural Sciences, Inter American University of Puerto Rico, San Juan, Puerto Rico, USA; f Grupo Infección y Cáncer, Facultad de Medicina, Universidad de Antioquia, Medellín, Colombia; g Departamento de Biología Celular, Facultad de Ciencias, Universidad Nacional Autónoma de México, México City, Mexico; h Department of Biological Sciences and Institute for Bioinformatics and Evolutionary Studies, University of Idaho, Moscow, Idaho, USA; i Department of Mathematics, University of Puerto Rico, San Juan, Puerto Rico, USA; j Anthropology Department, London School of Economics, London, England; k Department of Medicine, New York University School of Medicine, New York, New York, USA; University of Michigan—Ann Arbor

**Keywords:** diversity, human papillomavirus, lifestyle, oncogenic virus, urbanization

## Abstract

Human papillomavirus (HPV), an etiological agent of cervical cancer (CC), has infected humans since ancient times. Amerindians are the furthest migrants out of Africa, and they reached the Americas more than 14,000 years ago. Some groups still remain isolated, and some migrate to towns, forming a gradient spanning urbanization. We hypothesized that, by virtue of their history, lifestyle, and isolation from the global society, remote Amerindian women have lower HPV diversity than do urban women (Amerindian or mestizo). Here we determined the diversity of the 25 most relevant cervical HPV types in 82 Amerindians spanning urbanization (low, medium, and high, consistent with the exposure to urban lifestyles of the town of Puerto Ayacucho in the Venezuelan Amazonas State), and in 29 urban mestizos from the town. Cervical, anal, oral, and introitus samples were taken, and HPVs were typed using reverse DNA hybridization. A total of 23 HPV types were detected, including 11 oncogenic or high-risk types, most associated with CC. Cervical HPV prevalence was 75%, with no differences by group, but Amerindians from low and medium urbanization level had significantly lower HPV diversity than mestizos did. In Amerindians, but not in mestizos, infections by only high-risk HPVs were higher than coinfections or by exclusively low-risk HPVs. Cervical abnormalities only were observed in Amerindians (9/82), consistent with their high HPV infection. The lower cervical HPV diversity in more isolated Amerindians is consistent with their lower exposure to the global pool, and transculturation to urban lifestyles could have implications on HPV ecology, infection, and virulence.

**IMPORTANCE** The role of HPV type distribution on the disparity of cervical cancer (CC) incidence between human populations remains unknown. The incidence of CC in the Amazonas State of Venezuela is higher than the national average. In this study, we determined the diversity of known HPV types (the viral agent of CC) in Amerindian and mestizo women living in the Venezuelan Amazonas State. Understanding the ecological diversity of HPV in populations undergoing lifestyle transformations has important implication on public health measures for CC prevention.

## Abstract (precis)

The role of HPV type distribution on the disparity of cervical cancer (CC) incidence between human populations remains unknown. The incidence of CC in the Amazonas State of Venezuela is higher than the national average. In this study, we determined the diversity of known HPV types (the viral agent of CC) in Amerindian and mestizo women living in the Venezuelan Amazonas State. Understanding the ecological diversity of HPV in populations undergoing lifestyle transformations has important implication on public health measures for cervical cancer prevention.

## INTRODUCTION

Cervical human papillomavirus (HPV) infection (1) is a viral infection of the cervical epithelium (2) and the cause of cervical cancer (CC). It is nearly totally sexually transmitted. More than 80% of sexually active women are infected at least once in their lifetime (3), and its prevalence in a population mostly depends on the multiplicity of sexual partners (4). The course of the infection leads to either clearance by the immune system or persistence as an episome in infected cells (5). More than 180 HPV types have been completely sequenced (http://pave.niaid.nih.gov) (6), and around 40 have mucosal tropism (7). The types of HPVs circulating in a population can be defined by geographical and biological interaction among different HPV types and host immunogenic characteristics (e.g., HLA polymorphisms) (8).

Cervical cancer is one of the five deadliest types of cancer among women. As high as 80% of CC cases occur in developing countries (9, 10), with high mortality due to lower preventive medical screening, higher infection by virulent types, or both. In Venezuela, CC is the main cause of female deaths by cancer (11), with an incidence of 29 per 100,000. In the Amazonas State, the incidence is even higher, of 46 per 100,000 (11), consistent with other reports in Amazonian Amerindians (12).

HPV prevalence among Venezuelan women with normal cytologies has been reported to be 22 to 37% (*N* = 238 and *N* = 409, respectively) (13), with seven HPV types detected, including 23% HPV18 and 15% HPV16, followed by HPV31, HPV52, HPV45, HPV58, and HPV56 (<0.5%) (14). Among Venezuelan CC patients, the most common types are 68% HPV16 and 12% HPV18 followed by HPV33, -45, -31, -35, -58, -52, -26, -53, and -66 (<6.3%) (13). One of the very few studies in Amerindians in Brazil reported a prevalence of 46% in a population with 5.6% cytological abnormalities with the most common types being HPV16, HPV31, and HPV18 (15).

The evolution of HPV diversity is not well-known, but HPV has infected humans since times that preceded the human migrations out of Africa (16, 17). Amerindian ancestors that populated the Americas 14,000 to 24,000 years ago (18, 19) must have carried HPVs. We hypothesized that, consistent with their isolation and smaller community sizes, traditional Amerindians from remote villages have lower HPV diversity than urban women do. In this work, we compared HPV diversity between Piaroa Amerindians (living in a gradient of urbanization, from rainforest to town) and town mestizos.

## RESULTS

**FIG 1:**
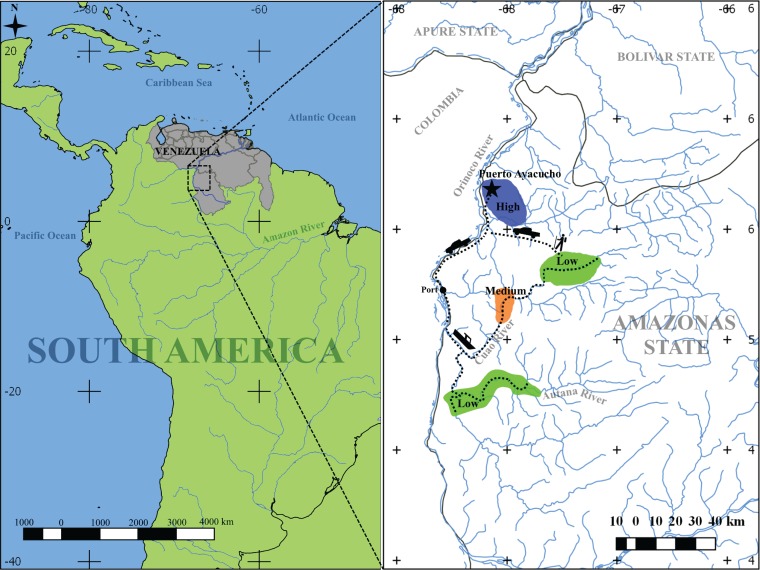
Diagram of geographic locations included in this study. Sampling was performed at eight locations with different urbanization levels: five locations with low urbanization level (green), one location with medium urbanization level (orange), and two locations with high urbanization (blue). Distances to the urban town were 150 to 210 km (by road and river) for the medium and low urban-level communities. Most communities can be reached only by river; however, some low-level urban communities can be accessed by 1 to 2 days of trekking through the forest. The medium urban level community is located 190 km from an urban location (130 km by river and 60 km by road). The two high urban level communities are located 8 km from each other. The map was generated using Quantum GIS Geographic Information System v. 2.18.14 (https://www.qgis.org/en/site/).

We determined the prevalence and diversity of HPV types in 111 sexually active women in the northern part of the Venezuelan Amazonas State in the Orinoco River basin (Fig. 1). The study included 82 Amerindians living in a spectrum of urbanization (defined as the gradient in lifestyle from traditional to urban), including 24 Amerindians living in traditional villages in the rainforest (low urbanization), 28 living in villages more exposed to non-Amerindians (medium urbanization level), and 30 living in the town capital of the Amazonas State, Puerto Ayacucho, which has a high mestizo population (high urbanization level). We also included 29 mestizos from the town. Surveys were applied to women to determine an individual (subject-based) or village (community-based) urbanization score (see Fig. S1 in the supplemental material; see also the data posted at https://doi.org/10.6084/m9.figshare.5579299.v1). Principal-component analysis (PCA) showed better segregation of subject-based groups (*P* < 0.003; Fig. S1c and e), than community-based classification (Fig. S1b and d; see also the data posted at https://doi.org/10.6084/m9.figshare.5579299.v1).

10.1128/mSphere.00176-18.1 FIG S1  Urban score assignment based on community or subject attributes. (a) Principal-component analysis (PCA) depicting villages based on urban scores. Five villages with low urbanization scores (green), one village with an intermediate score (orange), and two villages with high scores (blue) are shown. Principal component 1 (PC1) separated by urbanization indicator (italic labels), in a gradient from low to high urban level. Urban attribute vectors show directions of community location in the space, with high urban communities placed in the direction of the vectors. The length of the arrow is proportional to the contribution of each urbanization indicator explaining the spatial distribution of women. (b and c) PCA depicting individual urban scores colored by urban groups based on their village (community-based groups) (b) or subject-based groups (c). Community-based medium urbanization and high urbanization groups overlapped their 95% confidence interval (CI_95%_) ellipses, while low urbanization Amerindians cluster apart from mestizo women (ID refers to Identification Document possession). Subject-based medium urbanization and high urbanization Amerindians groups showed less overlap. The area of each ellipse represents each group distribution with the CI_95%_. (d and e) Urban index boxplots for community-based groups (d) and subject-based groups (e). Mean urban index comparison indicated significant increase from low to high urbanization Amerindian groups and with mestizos being the highest for both classification approaches (*P* = 0.000 by ANOVA; *P* < 0.003 by Tukey’s test for all paired comparisons). Different letters over the boxplots indicate significant differences. *P* values shown were still significant after Holm correction. (f) Distribution of women by their subject-based urban group (color legend), in each of the community-based urban groups (*x* axis). The dominant color in each community-based group was concordant with the subject-based urban group, the discordant cases showed heterogeneity within communities reflecting an urbanization transition that occurs at the subject level. (g and h) Linear regression of community versus subject urbanization indices including only Amerindians (g) and for all subjects (66 Amerindians and 24 mestizos) (h). A positive correlation was observed for both cases (linear model, *r*^2^ = 0.73, *y* = 0.636*x* + 0.123, and slope, *P* = 0.000 and *r*^2^ = 0.56, *y* = 0.477*x* + 0.196, slope, *P* = 0.000, respectively). Download FIG S1, PDF file, 0.9 MB. Copyright © 2018 Vargas-Robles et al. 2018 Vargas-Robles et al. This content is distributed under the terms of the Creative Commons Attribution 4.0 International license.

On the basis of the surveys (see the data posted at https://doi.org/10.6084/m9.figshare.5579299.v1), 77% of the women in the study had never had a cytological screening before. There were no age differences by urbanization level (mean, 28.9 years), use of hormonal contraceptives (uncommon in all groups), or lifetime sexual partners (Table 1 and Table S1). As expected, intestinal protozoa and helminthes were more prevalent in Amerindians than in mestizos (Table  2 and Table S2), and there was a significant increase in Amerindian schooling, sexual contact with mestizo men, smoking, and reduction in parity (number of times a woman has given birth), with urbanization (Table 1 and Table S1). Amerindian women reported practicing only vaginal intercourse, while 28% of mestizo women reported additional practice of oral and/or anal sex.

10.1128/mSphere.00176-18.4 TABLE S1  Demographic characteristics, contraception, and sexual behavior for 111 women of the study by community-based urbanization groups. Download TABLE S1, PDF file, 0.1 MB. Copyright © 2018 Vargas-Robles et al. 2018 Vargas-Robles et al. This content is distributed under the terms of the Creative Commons Attribution 4.0 International license.

10.1128/mSphere.00176-18.5 TABLE S2  Cervical HPV prevalence, cytological results, and prevalence of intestinal helminthes and anemia by community-based urbanization groups. Download TABLE S2, PDF file, 0.1 MB. Copyright © 2018 Vargas-Robles et al. 2018 Vargas-Robles et al. This content is distributed under the terms of the Creative Commons Attribution 4.0 International license.

**TABLE 1:** Demographic characteristics, condition, contraception use, and sexual behavior for 91 women^a^

Variable^b^	Value of variable for:				*P* value^c^	
	Amerindians in the following urbanization group:			Mestizos		
	Low	Medium	High		Amerindians from urbanization groups	Amerindians high vs mestizos
No. of subjects	22	22	23	24	1.000 (a)	1.000 (a)
Age (yr), mean [range]	31.1 [12–46]	31.3 [18–42]	29.2 [18–44]	26.7 [17–53]	0.930 (b)	0.320 (b)
Educational level (%) (*n*/*N*) [95% CI]						
No studies	68.2 (15/22) [45.1–85]	13.6 (3/22) [3.6–34]	4.3 (1/23) [0.3–24]	4.1 (1/24) [0.2–23]	1 × 10^−6^* (a)	1.000 (a)
Finished elementary school only	31.8 (7/22) [15–54]	50.0 (11/22) [31–69]	17.3 (4/23) [5.7–40]	16.7 (4/24) [5.5–38]	0.080 (a)	1.000 (a)
Finished high school	0.0 (0/22) [0–18.4]	36.4 (8/22) [18–59]	78.3 (18/23) [56–92]	83.3 (20/24) [62–95]	3 × 10^−8^* (a)	0.730 (a)
Currently using hormonal contraceptive^d^ (%) (*n*/*N*) [95% CI]	4.5 (1/22) [0.2–25]	0.0 (0/22) [0.0–19]	4.3 (1/23) [0.2–24]	29.2 (7/24) [13–51]	0.600 (a)	0.060 (a)
Parity, mean no. [range]	5.1 [0–11]	4.6 [0–13]	2.0 [1.0–6]	1.8 [0–8]	0.003* (b)	0.730 (b)
Currently breastfeeding (%) (*n*/*N*) [95% CI]	72.7 (16/22) [49–88]	50 (11/22) [31–69]	39.1 (9/23) [20–61]	70.8 (17/24) [49–87]	0.071 (b)	0.059 (b)
Median no. of sexual partners in sexual history [range]	2.0 [1–4]	2.5 [1–6]	2.0 [1–15]	2.0 [1–25]	0.850 (c)	0.210 (c)
No. of sexual partners in last 60 days (%) (*n*/*N*) [95% CI]						
None	22.7 (5/22) [8.7–46]	27.3 (6/22) [12–50]	30.4 (7/23) [14–53]	16.7 (4/24) [5.5–38]	0.840 (a)	0.490 (a)
1	77.2 (17/22) [54–91]	72.7 (16/22) [50–88]	69.6 (16/23) [47–86]	79.2 (19/24) [58–92]		
Weekly sexual intercourse frequency (%) (*n*/*N*) [95% CI]						
≤1 times	91.0 (20/22) [69–98]	72.7 (16/22) [50–88]	69.6 (16/23) [47–86]	41.7 (10/24) [23–63]	0.180 (a)	0.100 (a)
≥2 times	9.1 (2/22) [16–31]	27.3 (6/22) [12–50]	30.4 (7/23) [14–53]	58.3 (14/24) [37–77]		
Sexual contact with mestizo (%) (*n*/*N*) [95% CI]	0.0 (0/22) [0.0–19]	22.7 (5/22) [8.6–46]	34.8 (8/23) [17–57]	100 (24/24) [83–100]	0.012 (a)	7 × 10^−6^* (a)
Currently smoking^e^ (%) (*n*/*N*) [95% CI]	0.0 (0/22) [0.0–19]	4.5 (1/22) [0.0–25]	8.6 (2/23) [1.5–30]	16.7 (4/24) [5.5–38]	0.768 (a)	0.484 (a)

^a^ Demographic characteristics, contraception use, sexual behavior, and other characteristics (variables) are compared for Amerindians in the three subject-based urbanization groups (low, medium, and high) and for urban mestizos.

^b^ *n*/*N* is the number of women with that characteristic/total number of women in that group. The values for 95% confidence interval (95% CI) are shown in brackets.

^c^ The *P* values comparing the values for Amerindians in the high urbanization group compared to the values for mestizos are shown in the rightmost column. The tests used are shown in parentheses after the *P* value as follows: (a), χ^2^ test or Fisher's exact test; (b), *t* test and ANOVA for two groups or more than two groups; (c), Kruskal-Wallis test. An asterisk indicates that significant differences were reached (*P* < 0.05) after Holm correction for multiple comparisons.

^d^ For nonhormonal contraceptive use, the values were as follows: for Amerindians, zero cases for the low urbanization group, one sterilization for the medium urbanization group, and two sterilizations and one condom use case for the high urbanization group; for mestizos, three condom use cases.

^e^ Smoking frequency from 1 to 10 cigarettes daily during 1 or more years.

**TABLE 2:** HPV prevalence, cytological results, intestinal helminthes, and anemia prevalence among subject-based urban groups

Variable	Value of variable for:				*P* value^a^	
	Amerindians in the following urbanization group:			Mestizos		
	Low	Medium	High		Amerindians from urban groups	Amerindians high vs mestizos
Prevalence (%) of any HPV type^b^ (*n*/*N*) [95% CI]	63.6 (14/22) [41–82]	68.2 (15/22) [45–85]	78.3 (18/23) [56–92]	79.2 (19/24) [57–92]	0.546	1.000
HPV^b^ prevalence (%) by age (*n*/*N*) [95% CI]						
≤35 years old	57.2 (8/14) [30–81]	69.0 (9/13) [39–90]	75.0 (12/16) [47–92]	75.0 (15/20) [51–90]	0.657	1.000
>35 years old	75.0 (6/8) [36–96]	67.0 (6/9) [31–91]	85.7 (6/7) [40–100]	100 (4/4) [40–100]	0.843	1.000
Prevalence (%) of any HPV type^b^ excluding women with cervical abnormality (*n*/*N*) [95% CI]	60 (12/20) [36–80]	61.1 (11/18) [36–82]	77.2 (17/22) [54–91]	86.6 (19/22) [64–96]	0.414	1.000
Prevalence (%) of any high-risk HPV type^b^ (*n*/*N*) [95% CI]	54.5 (12/22) [33–75]	68.2 (15/22) [45–85]	78.3 (18/23) [56–92]	62.5 (15/24) [41–80]	0.237	0.389
Prevalence (%) of multiple HPV types^c^ among HPV-positive women (*n*/*N*) [95% CI]	71.4 (10/14) [42–90]	66.7 (10/15) [39–87]	38.9 (7/18) [18–64]	61.2 (11/19) [36–82]	0.124	0.408
Prevalence (%) of cervical abnormalities (*n*/*N*) [95% CI]	9.1 (2/22) [1.6–31]	18.2 (4^g^/22) [6.0–41]	4.3 (1/23) [0.2–24]	0.0 (0/22^d^) [0.0–15]	0.287	0.489
Prevalence (%) of cervical inflammation (*n*/*N*) [95% CI]	100 (22/22) [82–100]	100 (22/22) [82–100]	95.7 (22/23) [76–100]	100 (22/22^d^) [82–100]	1.000	1.000
Prevalence (%) of intestinal helminthes^e^ (*n*/*N*) [95% CI]	75 (15/20) [51–90]	65 (13/20) [41–84]	33.3 (5/15) [13–61]	28.6 (2/7) [5.1–70]	0.038	1.000
Prevalence (%) of anemia^f^ (*n*/*N*) [95% CI]	27.3 (6/22) [12–50]	27.3 (6/22) [12–50]	13.0 (3/23) [3.4–35]	0.0 (0/24) [0.0–17]	0.415	0.218

^*a*^The *P* values comparing the values for Amerindians in the high urbanization group compared to the values for mestizos are shown in the rightmost column. *P* value reached significant differences (*P* < 0.05) after Holm correction for multiple comparisons. The χ^2^ test or Fisher’s exact test was used.

^b^ High-risk HPV detected by the LiPA25 test: HPV types 16, 18, 31, 33, 35, 39, 45, 51, 52, 56, 58, and 59. Low-risk HPV detected by the LiPA25 test: HPV types 6, 11, 34, 40, 42, 43, 44, 53, 54, 66, 68/73, 70, and 74. Note that any incidence in type 68/73 is counted as one HPV type.

^c^ More than one HPV from any risk type.

^d^ Two cytology results from mestizo group were excluded because of poor-quality smears.

^e^ Ascaris lumbricoides, Hymenolepis diminuta, Trichuris trichiura, Enterobius vermicularis, Strongyloides stercoralis, and *Ancylostomatidae*.

^f^ Hemoglobin levels lower than 120 (grams/liter), according to the WHO.

^g^ One woman was negative by cytology but positive by biopsy specimen.

**FIG 2:**
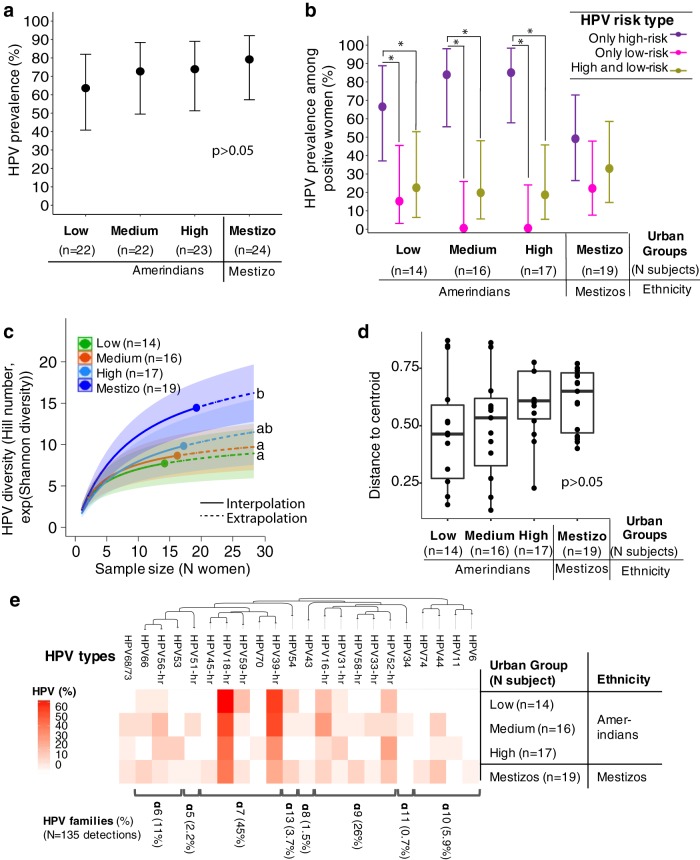
Prevalence and diversity of cervical HPV by subject-based urban groups. (a) HPV general prevalence. (b) HPV risk type prevalence. No prevalence differences were found among Amerindian groups (*P* = 0.540 by *χ*^*2*^ test) or between Amerindians from the high urban group and mestizos (*P* = 1.000 by *χ*^*2*^ test). Unlike mestizos, Amerindian women showed higher prevalence of only high-risk HPV types in relation to low-risk HPV or both types (*P* = 0.007 in the log linear model). The circles represent mean prevalence, and the bars show 95% confidence intervals (95% CIs). Prevalence that is statistically significantly (*P* < 0.050) different is indicated by a bar and asterisk. (c) Shannon diversity (Hill number *q* = 1) of cervical HPV by urban groups, based on a rarefied/extrapolated sample size of 28 women. Amerindians for low and medium urban groups were significantly less diverse than mestizos. There was a nonsignificant tendency to increasing HPV diversity with urbanization. The solid line curve fraction (interpolation) corresponds to the actual number of women sampled. The dashed line corresponds to the estimated diversity (extrapolation). Curved shaded areas represent the 95% CIs estimated from the bootstrap (50 replications). Significant differences are reached when 95% CIs do not overlap. Different letters indicate significant differences. (d) Beta diversity analysis by urban groups. Median distance to the centroid using Sorensen dissimilarity index. No difference among or within a group’s dispersion was observed (*P* > 0.05, PERMANOVA and permutation test for homogeneity of multivariate dispersions). (e) Heat map of prevalence of cervical HPV types. HPV18 and HPV39 of the α7 family showed the highest relative proportions. HPV L1 region sequences were used to generate a maximum likelihood tree rooted with theta HPV type (not shown). HPV families and their relative proportions (as a percentage; among only HPV-positive samples) are shown on the right. HPV68 and HPV73 were excluded from the tree, since the LiPA25 kit does not discriminate between these two types.

The overall prevalence of cervical HPV in this study was 75% (74%, excluding cervical abnormalities; see below) and did not differ between urban groups (*P* > 0.05; Table 2, Table S2, Fig. 2a, and Fig. S2a). There was a median of 1 to 2 HPV types per woman (Table 3 and Table S3; not different between groups; *P* > 0.05), and the differences in the frequency of single or multiple HPV infections were not significant between groups (*P* > 0.124; Table 2 and Table S2). In Amerindians, but not in mestizos, the prevalence of infections by exclusively high-risk HPVs was higher than infections with exclusively low-risk HPVs or with both HPV risk types (*P* = 0.007; Fig. 2b and Fig. S2b).

10.1128/mSphere.00176-18.2 FIG S2  Prevalence and diversity of cervical HPV by community-based urban groups. (a) HPV general prevalence. (b) Risk type prevalence. No general differences were found in prevalence by Amerindian groups (*P* = 0.540 by χ^2^ test) or between Amerindians from the high urbanization group and mestizos (*P* = 0.570 by χ^2^ test). Unlike mestizos, Amerindian women showed higher prevalence of having only high-risk HPV types in relation to low-risk HPV types or both types (*, *P* < 0.05, log-linear model). Dots represent prevalence, and bars are 95% confidence interval (CI_95%_). (c) Sample size-based Shannon diversity of cervical HPV by urban groups on an extrapolated 32 women. Amerindians for low and high urbanization groups were significantly less diverse than the mestizo group. There was a nonsignificant tendency of increasing HPV diversity with urbanization (CI_95%_, *P* > 0.05). The interpolation (solid line) curve fraction corresponds to the HPV diversity of the actual number of women sampled. The extrapolation fraction (dashed lines) corresponds to the estimated diversity. Curved shaded areas represent the CI_95%_ estimated from the bootstrap (50 replications). Different letters indicate significant differences, reached when CI_95%_ do not overlap. (d) Beta diversity analysis by urban groups. Median distance to centroid, using Sorensen dissimilarity index. No significant difference in variability between or within groups was observed (*P* > 0.05 by PERMANOVA and permutation test for homogeneity of multivariate dispersions). (e) Heat map of the prevalence of cervical HPV types. HPV18 and HPV39 of the α7 family show the highest relative proportions. HPV L1 region sequences were used to generate a maximum likelihood tree rooted with theta HPV type (not shown). HPV families and their relative proportions (as a percentage; among only HPV-positive samples) are shown on the right. HPV68 and HPV73 were excluded from the tree, since the LiPA25 kit does not discriminate between these two types. Download FIG S2, PDF file, 0.8 MB. Copyright © 2018 Vargas-Robles et al. 2018 Vargas-Robles et al. This content is distributed under the terms of the Creative Commons Attribution 4.0 International license.

10.1128/mSphere.00176-18.6 TABLE S3  Cervical HPV alpha and gamma diversities among HPV-positive women by community-based urbanization groups. Sample size-based, coverage-based, and asymptotic diversity analyses were performed for observed richness and Shannon and Simpson diversity (Hill numbers of order *q* = 1, 2, and 3, respectively) at a rarefied/extrapolated sample size of 32 women or 83 women for all populations (gamma diversity) or at a coverage-based level of 0.937 and 0.977, respectively. Shannon and Simpson metrics show mestizo women with a significantly higher HPV diversity than low urbanization Amerindians. Mestizos had a significantly higher Simpson index than any other Amerindian group in the asymptotic estimation. There was a nonsignificant tendency of increasing diversity with urbanization in Amerindians. Download TABLE S3, PDF file, 0.1 MB. Copyright © 2018 Vargas-Robles et al. 2018 Vargas-Robles et al. This content is distributed under the terms of the Creative Commons Attribution 4.0 International license.

**TABLE 3:** Cervical HPV alpha, beta, and gamma diversity measures^a^

Diversity measure	Value for diversity measure for the following^b^:				
	Amerindians in the following urbanization group:			Mestizos (*n* = 19)	All individuals (*n* = 66)
	Low (*n* = 14)	Medium (*n* = 16)	High (*n* = 17)		
Median no. of HPV types per woman [range]^c^	2 [1.0–4.0]	2 [1.0–4.0]	1 [1.0–4.0]	2 [1.0–6.0]	2 [1.0–6.0]
No. of high- and low-risk HPV types^d^	11	12	13	18	21
No. of high-risk HPV types^d^	7	8	10	11	11
No. of low-risk HPV types^d^	5	5	2	7	10
Observed richness (Hill no. *q* = 0) [95% CI]	13.2 [8.7–17.7] (A)	13.7 [9.9–17.6] (A)	15.3 [11.5–19.2] (A)	19.7[16.0–23.4] (A)	21.0 [21.4–39.6]
Shannon diversity^e^ (Hill no. *q* = 1) [95% CI]	8.6 [6.0–11.3] (A)	9.4 [6.6–11.4] (A)	10.9 [8.2–13.7] (AB)	15.5 [11.4–19.6] (B)	12.6 [13.6–16.0]
Simpson diversity^e^ (Hill no. *q* = 2) [95% CI]	6.2 [4.1–8.4] (A)	7.0 [4.1–9.4] (AB)	8.2 [4.8–11.6] (AB)	12.4 [8.8–15.9] (B)	8.7 [8.7–10.9]
Mean Sorensen dissimilarity index^f^	0.755	0.757	0.819	0.826	

^a^ Alpha diversity analysis by urban groups was performed at a rarefaction/extrapolation of 28 women per group and at 66 women among all population (gamma diversity).

^b^ The presence of different capital letters within parentheses across groups indicate significant differences based on the non-overlapping of their 95% CI in brackets.

^c^ Median comparison was performed with Kruskall-Wallis test. Two comparisons were performed: among Amerindian groups and between Amerindians from high urbanization and mestizos; none were statistically significant.

^d^ High-risk HPV detected by the LiPA25 test: HPV types 16, 18, 31, 33, 35, 39, 45, 51, 52, 56, 58, and 59. Low-risk HPV detected by the LiPA25 test: HPV types 6, 11, 34, 40, 42, 43, 44, 53, 54, 66, 68/73, 70, and 74. Note that any incidence of 68/73 is counted as one HPV type.

^e^ Shannon diversity refers to exp(Shannon diversity), and Simpson diversity refers to 1/Simpson index.

^f^ Sorensen index of dissimilarity. Comparisons were performed with permutation test for homogeneity of multivariate dispersions, based in 99 permutations. No group was significantly different.

A total of 23 HPV types were detected, of which 22 were from the cervix (Table S3). Alpha diversity was significantly higher in mestizos than in Amerindians from the lowest urban levels (based on subject-based groups in Fig. 2c, Table 3, and Table S4; community-based classification in Table S3 and Fig. S2c). The group differences in alpha diversity were mainly due to relative abundance rather than richness of HPV types (Table 3 and Tables S3 and S4). Beta diversity analysis using Sorensen dissimilarity index showed a nonsignificant tendency of increasing with urbanization (Fig. 2d, Fig. S2d, Table 3, and Table S3). For both classification approaches (subject- and community-based urban groups), a hierarchical tree showed that mestizos segregated from Amerindian low and medium urban groups (see the data posted at https://figshare.com/s/9bffb3ea746016f78b4e).

10.1128/mSphere.00176-18.7 TABLE S4  Cervical HPV alpha, beta, and gamma diversities among positive women by subject-based urbanization groups. Coverage-based and asymptotic diversity analyses were performed for observed richness and Shannon and Simpson diversity (Hill numbers of order *q* = 1, 2, and 3, respectively). Coverage-based analyses for urban groups and mestizos were performed at a coverage level of 0.906, while for the total population, the coverage level was 0.971. Mestizos showed significant higher diversity than Amerindian low urbanization group. At the asymptotic estimation, mestizos showed a significant higher HPV diversity than the Amerindian low urbanization group. There was a nonsignificant tendency of increasing diversity with urbanization in Amerindians. Download TABLE S4, PDF file, 0.1 MB. Copyright © 2018 Vargas-Robles et al. 2018 Vargas-Robles et al. This content is distributed under the terms of the Creative Commons Attribution 4.0 International license.

The most common HPV family was α7, followed by α9. HPV18 and HPV39 were the most prevalent cervical types (Fig. 2e and Fig. S2e). Only six cervical HPVs, all of them high-risk types, were shared among women in the four groups (Fig. S3). A comparative analysis of body site HPV distribution in 16 women with at least one body site positive for HPV showed 15 viral types in the cervix (14 women), 6 in the introitus (10 women), 4 anal (7 women), and 7 oral (6 women) (Table S5). The highest HPV prevalence and diversity was found in the cervix (*P* < 0.050; Table S5), and cooccurrence of any high-risk HPV or HPV18 in different body sites was low (Cohen’s kappa coefficients of ≤0.26 and ≤0.37, respectively; Table S6).

10.1128/mSphere.00176-18.3 FIG S3  Prevalence of high- or low-risk cervical HPV types among infected women by urbanization groups. HPV18 and HPV39 showed the highest prevalence. Percentages were calculated per urbanization group. Download FIG S3, PDF file, 0.3 MB. Copyright © 2018 Vargas-Robles et al. 2018 Vargas-Robles et al. This content is distributed under the terms of the Creative Commons Attribution 4.0 International license.

10.1128/mSphere.00176-18.8 TABLE S5  HPV prevalence and alpha and beta diversity by body site in 18 women, of which 16 are HPV infected in at least one body site. Sample-size-based, coverage-based, and asymptotic diversity analyses were performed for observed richness and Shannon and Simpson diversity (Hill numbers of order *q* = 0, 1, and 2, respectively) at a rarefied/extrapolated sample size level of 12 women or at a coverage level of 0.854. The cervix had the highest observed richness. Beta diversity with Sorensen dissimilarity index did differ significantly between groups. Download TABLE S5, PDF file, 0.1 MB. Copyright © 2018 Vargas-Robles et al. 2018 Vargas-Robles et al. This content is distributed under the terms of the Creative Commons Attribution 4.0 International license.

10.1128/mSphere.00176-18.9 TABLE S6  Congruence of detection of any high-risk HPV or HPV18 across body sites. There was low to no significant agreement in the presence of high-risk HPV in different body sites. Agreement between body sites varied from “less than chance” to “fair.” The highest specificity was observed in any high-risk HPV cooccurrence in anal and introitus sites. The highest sensitivity was observed in HPV18 cooccurrence in cervix and introitus sites. Download TABLE S6, PDF file, 0.1 MB. Copyright © 2018 Vargas-Robles et al. 2018 Vargas-Robles et al. This content is distributed under the terms of the Creative Commons Attribution 4.0 International license.

Nine women presented cervical abnormalities, and they were all Amerindians with mostly high-risk HPV infections (Table 2, Table S2, and Table S7; see the data posted at https://doi.org/10.6084/m9.figshare.5579299.v1). The presence of cervical lesions in these women did not significantly change HPV diversity.

10.1128/mSphere.00176-18.10 TABLE S7  HPV types, age, and urban group classification of nine women with cervical abnormalities. Download TABLE S7, PDF file, 0.1 MB. Copyright © 2018 Vargas-Robles et al. 2018 Vargas-Robles et al. This content is distributed under the terms of the Creative Commons Attribution 4.0 International license.

## DISCUSSION

Urban groups segregated better using subject-based rather than community-based metrics of classification, likely because villages in transition are heterogeneous in the lifestyles of their individuals. Interestingly, the Amerindian women who lived in the town did not reach the high urban scores of mestizo women, reflecting a certain level of attachment to their traditional lifestyles.

Cervical HPV prevalence in this study is similar to that reported in high-risk populations (20, 21) and higher than in other reports that used the same detection method, in Latin America (37 to 51%) (20, 22), Europe (<20%) (23), and Japan (<20%) (24). This disparity in prevalence may be due to the high prevalence of anemia and intestinal helminthes, which may reduce HPV clearance (25), degree of isolation from the global HPV pool, etc., while for industrialized countries, vaccination is significantly reducing HPV infection (26, 27).

The results of more isolated Amerindians having lower HPV diversity than mestizos confirmed our hypothesis and are consistent not only with the Amerindian’s higher isolation from the global viral pool but also with their lower genetic diversity. Amerindians descend from Asians who migrated east from Africa in successive genetic bottlenecks, thus only a fraction of the population—and gene pools—advanced (19, 28). Across the urbanization gradient, Amerindians become more exposed to mestizos and increase their genetic diversity (mestizaje) as well as their exposure to the global viral pool. However, a previous study in isolated Yanomamis of Brazil (15) reported higher HPV diversity than in more urbanized Macuxi and Wapishana Amerindians. This contradiction might be affected by the HPV detection methods used or by the degree of real isolation of the studied populations. In this study, we used a sensitive hybridization method that recognizes 25 HPV types (29, 30) based on L1 gene, the most conserved region in the HPV genome (31). The sensitivity of the detection method could decrease if there was divergence of the HPV during the isolation of Amerindian groups in the last 12,000 to 24,000 years. However, the probability of new diversity seems low, based on the estimated 200,000 years of evolution for intratypic variation of HPV18 (32). The question of novel variants in Amerindians is beyond the scope of the present study that aimed at characterizing the known HPV types, but future metagenomic studies should address this important question.

In relation to the presence of HPV in multiple body sites, our study shows 33% oral HPV (which is higher than in other reports using the same detection method; e.g., 1.6% in Costa Rica [33]) and 30% anal prevalence (similar to that in other reports [22]). There was low cooccurrence of specific HPV types in different body sites, which might result from epithelial tropism (34, 35) or site-related clearance (36) and may depend on sexual practices, such as nonvaginal sex (37), that are uncommon in our studied population. However, there can be extrasexual HPV transmission, such as self-transmitted to different body sites, or mother-child vertical transmission (38). The fact that the introitus site showed lower HPV prevalence than cervix (24 versus 75%, respectively) has implications when self-sampling is used for sample collection in population cervical HPV screenings.

Understanding the causes underlying the high incidence of CC in Amerindians is of crucial importance for decisions in public health interventions. While the same virulent types circulate among Amerindian and mestizo women, Amerindians showed higher prevalence of infections by the virulent types than infection by low-risk types or both. Amerindians in this study did show high HPV18 and HPV16, common virulent types in other human groups, but they also had high prevalence of a rare high-risk HPV type of the α7 family, HPV39, consistent with reports for Amerindians in the northern United States (39) and Central and South America (40). Its prevalence in this study shows a nonsignificant trend to decrease with urbanization. Regrettably, contemporary HPV vaccines do not include this virulent HPV39 highly prevalent in these populations.

That cervical abnormalities were found only in Amerindians, consistent with the epidemiological evidence of high CC incidence in this human group (12), suggests that infections by only oncogenic HPVs increase the risk of cervical abnormalities; this was reported before for squamous CC (41). Amerindian genetic variations in the immune-relevant HLA-B locus may also increase their susceptibility to colonization by oncogenic types (42, 43). A high prevalence of only oncogenic HPV infections is consistent with the more efficient clearance of low-risk HPVs in relation to high-risk HPVs, which evade immune clearance, producing low virion yields (44, 45), and thus, the factors that sustain the coexistence of different HPV risk types in mestizos are unclear. Coexistence of high- and low-risk HPVs has been associated with higher sexual partner turnover (46), although we did not find differences in the reported number of sexual partners. Definitely, more studies are needed to clarify the relative contribution of lifestyle and host genetic factors to the type of HPV infection and health risks. The results of this study are consistent with the association between high-risk HPVs and increased inflammation and risks of cervical lesions (41, 47), and this is particularly serious in regions with precarious or nonexistent health services (48). Finally, the elimination of high-risk HPV types with the current vaccines is a promising scenario to reduce the dramatically high CC mortality in Venezuelan Amerindians. Studies that follow up the effects of the vaccines on the circulating HPV diversity, using metagenomic approaches (15) will be important for monitoring the evolution of HPV type virulence.

## MATERIALS AND METHODS

### Experimental design.

This study included young adult, nonpregnant, healthy women from the Venezuelan Amazon. The women were from the following two groups: Piaroa Amerindian from villages in a spectrum of urbanization (from traditional to urban lifestyles) or urban mestizo. All experimental protocols were approved by SA Centro Amazónico de Investigación y Control de Enfermedades Tropicales Simón, Bolívar, Venezuela (SACAICET, IRB 78-2014), and University of Puerto Rico (IRB 1314-163).

### Inclusion criteria.

Women included in the study belonged to eight different villages in northern Amazonas State, Venezuela: one urban town, Puerto Ayacucho (state capital), one village in the periurban area, and six villages at the Orinoco Basin on the Sipapo River, Autana River, and Cuao River (Fig. 1). A total of 228 sexually active women attending a health evaluation were invited to participate, and 111 (82 women who self-identified as Amerindians with Piaroa ethnicity, appeared to be Piaroa Amerindians, and also spoke Piaroa language and 29 urban mestizos) aged 12 to 53 years were included in the study. We had received prior approval from the captain/leader to visit the villages. Informed consent was obtained from all participants and/or their legal guardians. Parental consent was requested for women less than 18 years old. Inclusion criteria included women at reproductive age who at the time of recruitment had none of the following: pregnancy, menses, bleeding in the last 24 h, sexually transmitted infection diagnosed in the last 2 months, antibiotics in the last month, vaginal douches in the last 24 h, sexual intercourse in the last 24 h, hysterectomy, diabetes, urinary incontinence, urinary tract infections, and HIV. Individuals excluded from the study (*n </it>=* 117) were mostly due to recent exposure to antibiotics or antiparasitic drugs (28%), menses (25%), postmenopausal (13%), pregnant (12%), urinary infections (8%), refusing to participate (4%), sexual contact in the last 24 h (3%), hysterectomy (2%), belonging to a different ethnicity (1%), diabetes (1%), and HIV (1%).

### Surveys and urban classification.

Each woman received two urbanization indices, one based on her individual exposure to urban practices (subject-based index) and another on her community urban level (community-based index) (see data posted at https://doi.org/10.6084/m9.figshare.5579299.v1). Subject-based surveys included education, identification document (ID) possession, purchasing power, preservation of traditional practices, frequency of mobility to urbanized towns, level of environmental exposure (drinking water treatment, use of shoes, etc.), use and acceptance of Western medicine, and level of adoption of nontraditional diets (see data posted at https://doi.org/10.6084/m9.figshare.5579299.v1). Community-based urbanization survey included access to health, urban services (electricity, telephone, gas, and water), political representation, education, salaries, and language command (Spanish-Piaroa). This village survey was completed with the community captains, schoolteachers, or health workers (see data posted at https://doi.org/10.6084/m9.figshare.5579299.v1).

Categorical variables of the urbanization surveys were transformed into numeric values ranked between 0 and 1, with 1 being the highest level of urbanization (also reflecting the loss of traditional practices). Each indicator component was equally weighted, and its values were averaged using arithmetic means. Community-based groups included 111 women, but subject-based groups included only 91 women due to missing data in the surveys. Urban groups had similar sample sizes (Table 1 and see Table S1 in the supplemental material). Community urban indices were categorized in three levels: low (scores below 0.33; *n* = 24 women), medium (scores of >0.33 and <0.66; *n* = 28 women), and high (scores above 0.66; *n* = 30 women). Subject-based urbanization groups were built first, sorting in ascending order individual women scores and then grouping them in tertiles: the first group corresponds to the low urban group (*n* = 22 women; scores of 0.22 to 0.37), the second group corresponds to the medium urban group (*n* = 22 women; scores of 0.40 to 0.55), and the third group corresponds to the high urban group (*n* = 23 women; scores of 0.56 to 0.77). Mestizo women had a high urban level by both classification approaches (*n* = 29 to 24, respectively) (scores of 0.70 to 0.93 for subject-based groups).

Clinical history, sexual behavior, contraceptive usage, and hygiene practices were also recorded in a separate clinical survey (see the data posted at https://doi.org/10.6084/m9.figshare.5579299.v1). Surveys were coded without personal identifiers.

### Samples.

Swabs were taken by specialized health personnel, from cervix/fornix (referred to as cervix in the text) (*N* = 111), introitus (*N* = 18), anal (*N* = 18), and oral (*N* = 18) sites. DNA was extracted using Power Soil DNA kit (Mo Bio Laboratories Inc.) according to the manufacturer’s instructions. The main concern about HPV detection methods is obtaining false-negative results, usually after not being able to extract/detect viral DNA in an HPV-positive sample. The Power Soil method involves an aggressive bead beating step and allowing good extraction of the viral DNA. Cervical smears were performed by an obstetrician-gynecologist using an endocervical brush and spatula, and biopsy specimens were taken and treatment was provided if indicated. Papanicolaou’s stain was performed for the cytological analysis. Results were reported according to Bethesda 2001 classification system. A drop of blood was taken from fingers for *in situ* hemoglobin (using *Easylife* rapid test in peripheral blood) to detect anemia according to the WHO limits (49). Sera were transported for HIV, syphilis, and hepatitis B and C detection, processed at the Public Health Center of Puerto Ayacucho, Amazonas State, Venezuela. Fecal samples were taken, preserved using iodine-formaldehyde, and microscopically analyzed for the presence of intestinal protozoa and helminthes by microscopic methods.

### HPV genotyping.

The approach used in this study, the SPF10 assay that amplifies 60 different known HPV strains with high sensitivity (29, 30) and hybridizes the SPF10 PCR product on the LiPA25, was limited to 25 of the most relevant and prevalent known genotypes. A reverse hybridization method SPF_10_-PCR-LiPA25 system, version 1 (Labo Biomedical Products, Rijswijk, The Netherlands, based on licensed Innogenetics technology) (50), was used to detect HPV and typing 25 of the most common mucosa HPV types (types 6, 11, 16, 18, 31, 33, 34, 35, 39, 40, 42, 43, 44, 45, 51, 52, 53, 54, 56, 58, 59, 66, 68/73, 70, and 74). Briefly, 65-bp biotinylated amplicons from the highly conserved L1 gene region were generated using SPF10 primers. Amplified fragments were hybridized with a strip with specific oligonucleotide probes for each of the 25 HPV types. Visualization was performed by adding streptavidin-conjugated alkaline phosphatase to the hybrids formed, yielding a dark precipitate in a particular strip area that determines the specific HPV type. Negative and positive controls were included. We confirmed results of the highly sensitive method for HPV detection using the SPF_10_ primers (29, 30), repeating a subsample of replicate swabs from 10 women. This is a study performed in a non-HPV-vaccinated population, since HPV vaccines have not been included in the national vaccination program in Venezuela.

### Statistical analysis.

Principal-component analysis (PCA) for the villages and for women based on their urbanization indicator values were performed with the *ggfortify* package (51) in *R* (52). To visualize the urban groups for both types of classification, 95% confidence interval (95% CI) ellipses were drawn for community-based and subject-based group distributions (Fig. S1). Mean comparisons among urban group scores were performed with analysis of variance (ANOVA) and Tukey’s test as a posthoc test (Fig. S1). Correlations between village- and subject-based urban scores among all populations and only including Amerindians were evaluated by a linear regression (Fig. S1).

Association between prevalence of HPV types, having only a high-risk or low-risk type or both risk types, and comparisons among single and multiple types and among body sites, were performed using log linear models and the *contrast* package (53) in *R* version 3.3.2 (52) to compute comparisons of the estimated regression coefficient. Comparisons of means with normal distribution (verified by QQ plot) were performed with ANOVA and Tukey’s test as a posthoc test, and between Amerindians from high urbanization group and mestizo group with Student’s *t* test. Means with nonnormal distribution were compared using the Kruskal-Wallis test. Proportion comparisons among groups were performed with Pearson’s chi-squared test (*χ*^*2*^ test) with Yates’ continuity correction if needed or Fisher exact test for count data. *P* values were adjusted for multiple comparisons by the Holm method (54) (Table 1 and Tables S1 and S2). *P* values of <0.05 were considered statistically significant.

High-risk HPV and HPV18 detection agreement across body site were measured for only 18 women using Cohen’s kappa coefficient (55, 56) and were interpreted as follows: Cohen’s kappa coefficient of <0, less than chance; 0.01 to 0.20, slight; 0.21 to 0.40, fair; 0.41 to 0.60, moderate; 0.61 to 0.80, substantial; and 0.81 to 0.99, almost perfect (Tables S5 and S6) (56, 57).

Phylogenetic trees were built based on the HPV L1 region from sequences obtained from the PaVE database (58); MAFFT was used for the nucleotide alignment (59). The maximum likelihood method was used in PhyMLb (60). The tree was rooted with theta HPV type (not shown in the figure) (Fig. 2e and Fig. S2). The hierarchical tree for HPV and urban groups or individual women was built using *hclust* function from the R base “stats” package by the Spearman method and was visualized together with a heat map plotted with *heatmap.2* from gplots (61) and RColorBrewer (62) from R packages.

Alpha and gamma HPV diversity were measured using three of the most typical used Hill’s family of diversity (63–65) numbers or the effective number of types, order (q) 0, 1, and 2 (HPV type observed richness, exponential of Shannon entropy index, and inverse of Simpson concentration index) integrating rarefaction (interpolation) and extrapolation curves following Hsieh et al. (66) approach using *iNEXT* package (66) in *R 3.3.2* version (52). Hill number of order 0 (*q* = 0) counts all HPV types present in each group, Hill number of order 1 (*q* = 1) can be interpreted as the number of common HPV types per group, and Hill number of order 2 (*q* = 2) can be interpreted as the number of dominant HPV types. Alpha diversities were compared using the nonasymptotic and asymptotic analysis for incidence type data. For the nonasymptotic analysis, we compared groups at the same sample size (sample size based) or at the same level of sampling coverage (sample coverage based). The latter measures the proportion of the total number of individuals that belong to the HPV type detected in the sample and has been shown to better evaluate the magnitude of the diversity differences among groups than the traditional sample size-based comparison (64). The asymptotic analysis allowed estimating diversity when the accumulation curves reach the asymptote guided by the Chao2 estimator. Comparisons among groups were performed at the extrapolated diversity values. The 95% CI was estimated from the bootstrap method based on 50 replications. Significant differences were reached when the 95% CIs among groups did not overlap (Fig. 2c, Fig. S2, Table 3, and Tables S3to S5).

Beta diversity was analyzed with the *vegan* (67) package in R (52). Beta diversity was measured using the nonparametric permutational multivariate analysis of variance (PERMANOVA) (68) to compared variance between groups with the variance within groups. Sorensen dissimilarity index matrix was built with *betadiver* function. The model calculates a pseudo F ratio that is tested for significance based on 999 permutations. A more robust analysis for within group dispersion (variance) comparison was performed with the permutation test for homogeneity of multivariate dispersions (10). The *betadisper* function was used to reduce the distances to the principal coordinate. The method computes the F statistic to compare median distances-to-centroids of each group. *P* value was generated with the *permutest* function based on 999 permutations. The plot function was used for the principal-coordinate analysis visualization (Fig. 2d, Fig. S2, and Tables S3 and S4).

Statistics and graphics were also performed using *reshape2* (69), *ggplot2* (70) and defaults *R 3.3.2* version functions (52). The map was generated using QGIS Geographic Information System 2.18.14 (71).

### Data availability.

Three data sets containing urbanization survey results and metadata and other data have been deposited in figshare at https://doi.org/10.6084/m9.figshare.5579299.v1.
