# Supplementary figures and images for: High Rate of Infection by Only Oncogenic Human Papillomavirus in Amerindians
Source: mSphere. 2018 May 2;3(3):e00176-18. doi: 10.1128/mSphere.00176-18 (PMC5932372; doi:10.1128/mSphere.00176-18)

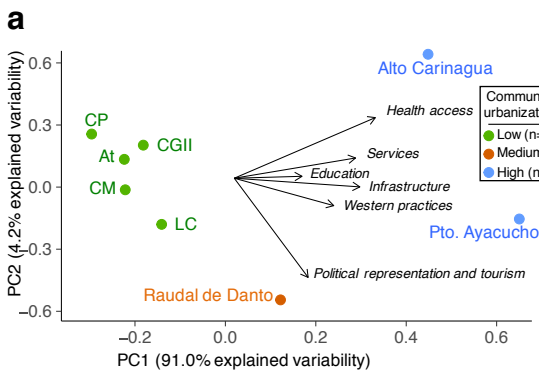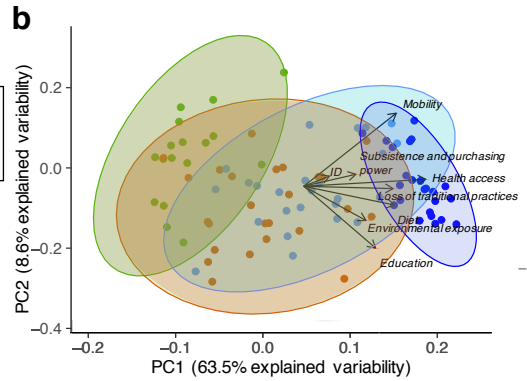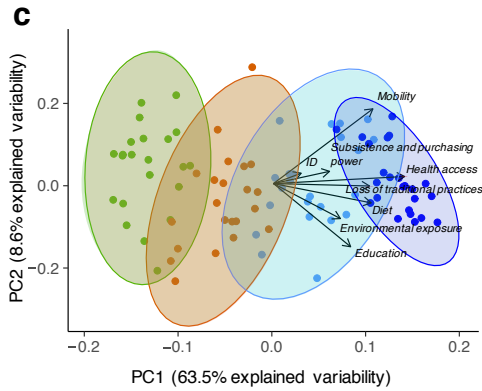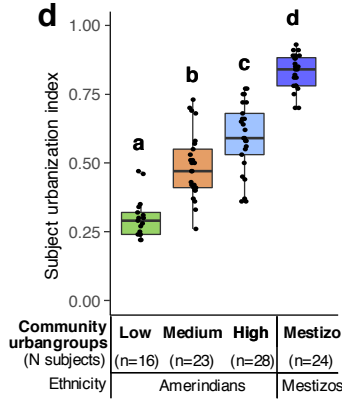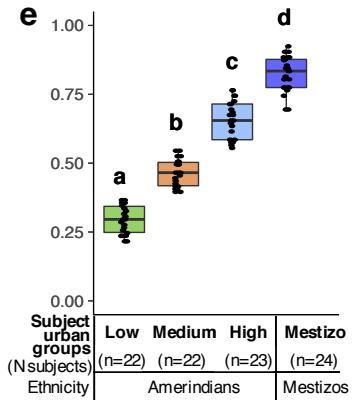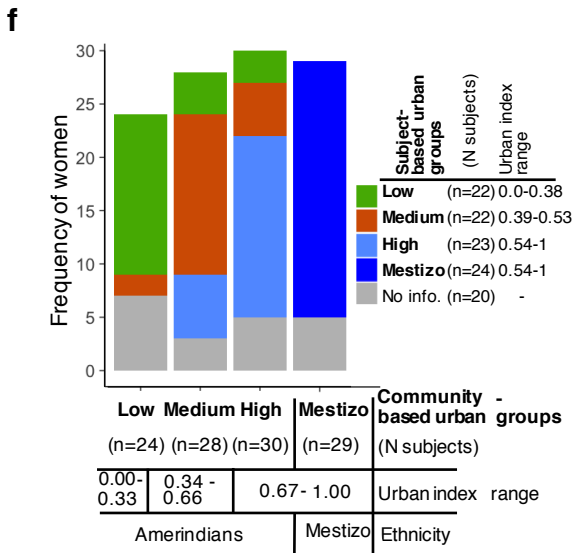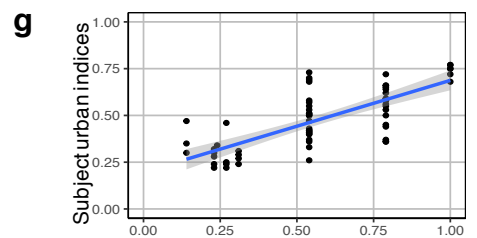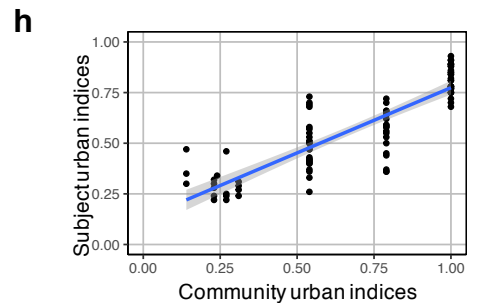

Supplement: FIG S1 [file sph003182535sf1.pdf]

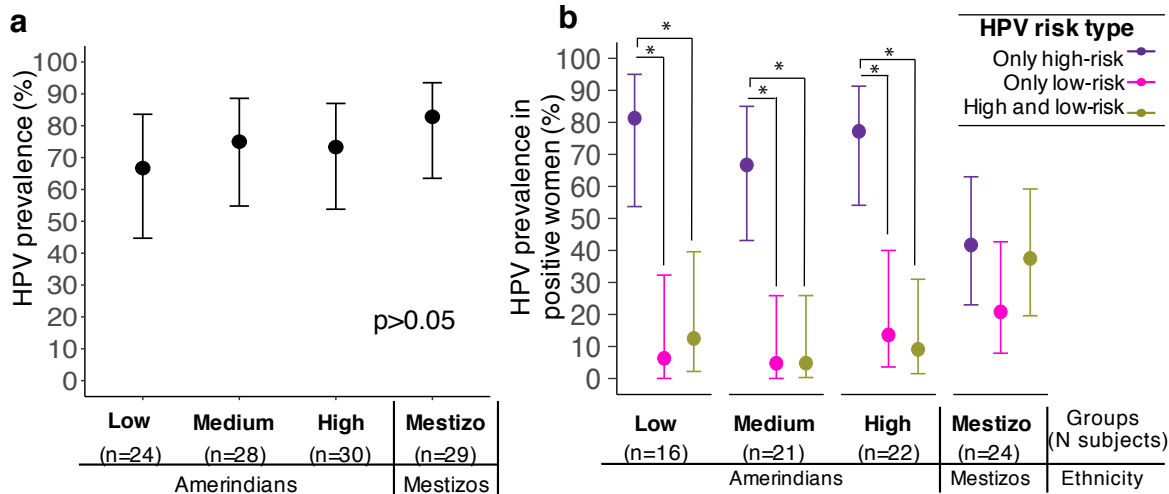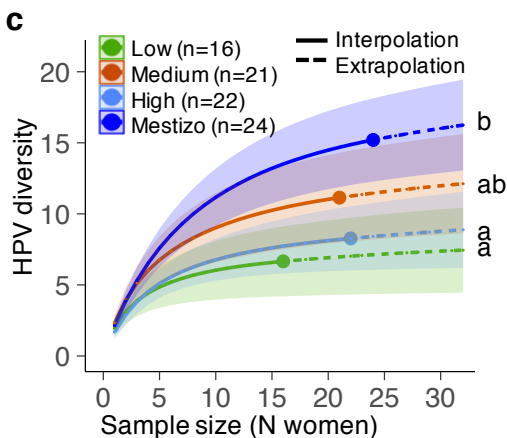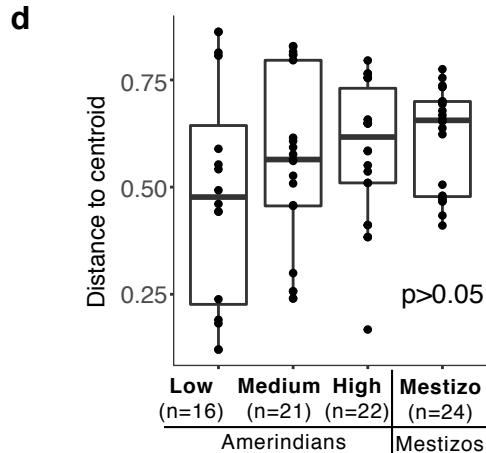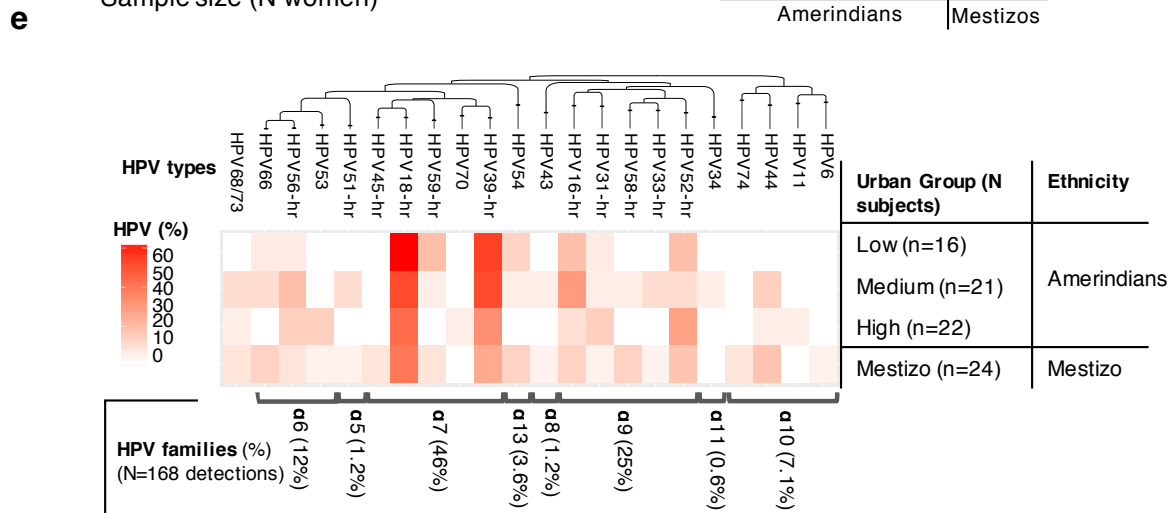

Supplement: FIG S2 [file sph003182535sf2.pdf]
