# Supplementary material for: High Rate of Infection by Only Oncogenic Human Papillomavirus in Amerindians
Source: mSphere. 2018 May 2;3(3):e00176-18. doi: 10.1128/mSphere.00176-18 (PMC5932372; doi:10.1128/mSphere.00176-18)
Supplement: TABLE S1 [file sph003182535st1.pdf]

Table S1

Variables	Amerindian community-based urban groups Low	Medium	High	Mestizos Mestizo	*p value* Amer-indians	Amer-indians high vs. Mestizos
**N subjects**	**24**	**28**	**30**	**29**	*0.710 (a)*	*0.890 (a)*
**Age, Mean [min, max]**	**28.3** [12-46]	**30.2** [18-42]	**31.2** [17-45]	**26.0** [17-53]	*0.500 (b)*	*0.025 (b)*
**Educational level % (n/N); [CI $_{95\%}$]**						
No studies	**64.7** (11/17); [41-86]	**16.0** (4/25); [5.3-37]	**16.0** (4/25); [5.3-37]	**4.1** (1/24); [0.2-23]	*0.001* (a)*	*0.350 (a)*
Finished elementary school	**23.5** (4/17); [7.8-50]	**40.0** (10/25); [22-61]	**32.0** (8/25); [16-54]	**12.5** (3/24); [3.3-33]	*0.570 (a)*	*0.170 (a)*
Finished high school	**11.8** (2/17); [2-39]	**64.8** (11/25); [37-85]	**52.0** (13/25); [32-72]	**83.3** (20/24); [62-95]	*0.020 (a)*	*0.220 (a)*
**Hormonal contraceptive** use currently % (n/N); [CI $_{95\%}$]**	**0.0** (0/24); [0-17]	**3.6** (1/28); [0.2-20]	**10.0** (3/30); [2.6-27]	**34.5** (10/29); [19-54]	*0.220 (a)*	*0.050 (a)*
**Parity, mean [IQR]**	**4.3** [0-11]	**4.0** [0-13]	**2.9** [0-12]	**1.8** [0-8]	*0.340 (b)*	*0.090 (b)*
**Breastfeeding currently % (n/N); [CI 95%]**	**62.5** (15/24); [41-80]	**62.9** (17/28); [41-78]	**36.7** (11/30); [21-56]	**70.0** (20/29); [49-84]	*0.094 (b)*	*0.026 (b)*
**Sexual partner history number, Median [min-max]**	**2.0** [1.0-10]	**2.0** [1.0-6.0]	**3.0** [1.0-15]	**2.0** [1.0-25]	*0.750 (c)*	*0.100 (c)*
**Number of sexual partners in last 60 days % (n/N); [CI $_{95\%}$]**					*0.750 (a)*	*0.260 (a)*
None	**25.0** (6/24); [11-47]	**21.4** (6/28); [9-42]	**30.0** (9/30); [15-50]	**13.8** (4/29); [4.5-33]		
1	**75.0** (18/24); [53-89]	**78.6** (22/28); [59-91]	**70.0** (21/30); [50-85]	**82.8** (24/29); [64-94]		
**Sexual intercourse weekly frequency % (n/N); [CI $_{95\%}$]**					*0.060 (a)*	*0.050 (a)*
≤ 1 time	**95.8** (23/24); [77-100]	**75.0** (21/28); [55-89]	**70.0** (21/30); [50-85]	**41.4** (12/29); [24-61]		
≥ 2 times	**4.2** (1/24); [0.2-23]	**25.0** (7/28); [11-45]	**30.0** (9/30); [15-50]	**58.6** (17/29); [64-94]		
**Sexual contact with mestizo % (n/N); [CI $_{95\%}$]**	**8.3** (2/24); [15-29]	**10.7** (3/28); [28-29]	**46.7** (14/30); [29-65]	**100.0** (29/29); [85-100]	*$6x10^{-4}$* (a)*	*$2x10^{-5}$* (a)*
**Currently smoking*** % (n/N); [CI $_{95\%}$]**	**0.0** (0/24); [0-17]	**0.0** (0/28); [0-15]	**10.0** (3/30); [2.6-27]	**13.8** (4/29); [4.5-33]	*0.034 (a)*	*0.699 (a)*

(a) X^2 test or Fisher's Exact Test

(b) T-test and ANOVA for 2 or more than two groups

(c) Kruskal-Wallis

*Significant differences reached (p <0.05) after Holm correction for multiple comparisons.

**For non hormonal contraceptive use, for Amerindians, Low group = 0 cases, Medium group = 1 sterilization, High group = 4 sterilizations and 2 condom use cases; for Mestizo = 3 condom use cases.

***Smoking frequency from 1 to10 cigarrettes daily during 1 or more years
