# Supplementary material for: High Rate of Infection by Only Oncogenic Human Papillomavirus in Amerindians
Source: mSphere. 2018 May 2;3(3):e00176-18. doi: 10.1128/mSphere.00176-18 (PMC5932372; doi:10.1128/mSphere.00176-18)
Supplement: TABLE S2 [file sph003182535st2.pdf]

Table S2

Variables	Amerindian community-based urban groups: Low	Amerindian community-based urban groups: Medium	Amerindian community-based urban groups: High	Mestizos: Mestizo	*p value**: *Amer-indians*	*p value**: *Amer-indians high vs. Mestizos*
**Any HPV type# % (n/N); [$CI_{95\%}$]**	**66.7** (16/24); [45-84]	**75.0** (21/28); [55-89]	**73.3** (22/30); [54-87]	**82.8** (24/29); [64-93]	*0.783*	*0.576*
**HPV prevalence# by age % (n/N); [$CI_{95\%}$]**						
≤35 years old	**61.1** (11/18); [36-82]	**70.6** (12/17); [44-87]	**79.0** (15/19); [54-93]	**80.0** (20/25); [59-92]	*1.000*	*0.527*
>35 years old	**83.3** (5/6); [37-99]	**81.8** (9/11); [47-97]	**63.6** (7/11); [32-88]	**100** (4/4); [40-100]	*0.632*	*0.517*
**Any HPV type#, excluding women with cervical abnormality % (n/N); [$CI_{95\%}$]**	**63.6** (14/22); [41-82]	**73.1** (19/26); [52-88]	**68.0** (17/25); [47-84]	**88.9** (24/27***); [64-93]	*0.780*	*0.254*
**Any high-risk HPV type# % (n/N); [$CI_{95\%}$]**	**62.5** (15/24)[41-80]	**71.4** (20/28)[51-86]	**66.7** (20/30)[47-82]	**65.5** (19/29); [46-81]	*0.790*	*1.000*
**Multiple HPV types** among positive women % (n/N); [$CI_{95\%}$]**	**56.3** (9/16); [79-31]	**66.7** (14/21); [85-43]	**40.9** (9/22); [63-22]	**62.5** (15/24)[80-41]	*0.234*	*0.242*
**Cervical abnormalities prevalence % (n/N); [$CI_{95\%}$]**	**8.3** (2/24); [1.4-29]	**7.1** (2/28); [1.2-25]	**16.7** (5/30&); [6.3-36]	**0.0** (0/27***); [0.0-16]	*0.505*	*0.053*
**Cervical inflammation % (n/N); [$CI_{95\%}$]**	**100** (24/24); [83-100]	**100** (28/28); [85-100]	**96.6** (28/29); [80-100]	**100** (27/27); [85-100]	*1.000*	*1.000*
**Intestinal helminthes&& % (n/N); [$CI_{95\%}$]**	**81.0** (17/21); [57-94]	**76.0** (19/25); [54-90]	**36.4** (8/22); [18-59]	**28.6** (2/7); [5.1-70]	*0.005**	*1.000*
**Anemia&&& (c) % (n/N); [$CI_{95\%}$]**	**41.7** (10/24); [23-63]	**21.4** (6/28); [9.0-42]	**16.7** (5/30); [6.3-36]	**3.4** (1/29); [1.6-20]	*0.092*	*0.211*

#High-risk HPV detected by the LiPA25 test: 16, 18, 31, 33, 35, 39, 45, 51, 52, 56, 58, 59. Low-risk HPV detected by the LiPA25 test: 6, 11, 34, 40, 42, 43, 44, 53, 54, 66, 68/73, 70, 74. Note: Any incidence in 68/73 is count as a one HPV type.

**p value* reached significant differences ($p<0.05$) after Holm correction for multiple comparisons. X^2 test or Fisher's Exact Test.

**More than one HPV from any risk type.

***Two cytology results from mestizo group were excluded because of poor quality smears.

&One woman was negative by cytology but positive by biopsy.

&&*Ascaris lumbricoides, Hymenolepis diminuta, Trichuris trichiura, Enterobius vermicularis, Strongyloides stercorali,* and Ancylostomatidae

&&&Hemoglobin levels lower than 120 (g/l), according to the WHO.
