# Supplementary material for: High Rate of Infection by Only Oncogenic Human Papillomavirus in Amerindians
Source: mSphere. 2018 May 2;3(3):e00176-18. doi: 10.1128/mSphere.00176-18 (PMC5932372; doi:10.1128/mSphere.00176-18)
Supplement: TABLE S3 [file sph003182535st3.pdf]

Table S3

Analysis	HPV diversity measures	Amerindian community-based urban groups Low (n=16)	Medium (n=21)	High (n=22)	Mestizos Mestizo (n=24)	All population (N=83)
Mean and HPV risk counts	HPV type median by woman [min-max]*	**2** [1.0-4.0]	**2** [1.0-5.0]	**1** [1.0-4.0]	**2** [1.0-6.0]	**2** [1.0-6.0]
		p>0.05				
	N of high and low-risk HPV types**	**9**	**16**	**11**	**18**	**22**
	N of high-risk HPV types **	**7**	**10**	**6**	**11**	**11**
	N of low-risk HPV types **	**2**	**6**	**5**	**7**	**11**
Sample size-based	Observed richness (Hill number ***q=0)*** [$CI_{95\%}$]	**10.2** [6.1-14.3] (a)	**16.7** [13.1-20.2] (ab)	**11.3** [8.3-14.3] (a)	**19.0** [15.6-22.3] (b)	**22.0** [22.4-40.7]
	Shannon diversity[&] (Hill number ***q=1***) [$CI_{95\%}$]	**7.1** [4.7-9.5] (a)	**11.4** [8.3-14.5] (ab)	**8.4** [5.8-10.9] (a)	**15.2** [11.7-18.7] (b)	**13.0** [13.0-16.0]
	Simpson diversity[&] (Hill number ***q=2)*** [$CI_{95\%}$]	**5.5** [3.6-7.5] (a)	**8.5** [6.6-10.3] (ab)	**6.8** [4.6-9.0] (a)	**12.3** [9.1-15.4] (b)	**9.0** [9.0-11.1]
Sample coverage-based	Observed richness (Hill number ***q=0)*** [$CI_{95\%}$]	**10.3** [6.4-14.2] (a)	**17.4** [13.2-21.6] (ab)	**14** [8.1-20.0] (ab)	**20.4** [16.3-24.6] (b)	**22.0** [19.1-24.9]
	Shannon diversity[&] (Hill number ***q=1***) [$CI_{95\%}$]	**7.1** [4.8-9.5] (a)	**11.8** [9.0-14.5] (ab)	**9.3** [6.2-12.5] (ab)	**16.2** [12.8-19.6] (b)	**12.9** [11.4-14.6]
	Simpson diversity[&] (Hill number ***q=2)*** [$CI_{95\%}$]	**5.6** [3.6-7.5] (a)	**8.6** [6.0-11.2] (ab)	**7.3** [4.9-9.7] (ab)	**13.0** [9.3-16.6] (b)	**9.0** [7.3-10.6]
Asymptotic estimation	Observed richness (Hill number ***q=0)*** [$CI_{95\%}$]	**13.2** [9.4-47.4] (a)	**19.4** [16.6-35.1] (a)	**18.6** [11.9-72.9] (a)	**22.4** [19.6-38.2] (a)	**24.6** [22.4-40.7]
	Shannon diversity[&] (Hill number ***q=1***) [$CI_{95\%}$]	**8.1** [6.6-10.9] (a)	**13.6** [11.1-17.2] (b)	**10.4** [8.2-14.3] (ab)	**19.1** [15.2-23.1] (b)	**14.0** [13.0-15.9]
	Simpson diversity[&] (Hill number ***q=2)*** [$CI_{95\%}$]	**5.9** [5.3-7.8] (a)	**9.4** [8.3-12.2] (b)	**7.7** [6.8-10.4] (ab)	**15.3** [12.3-19.5] (c)	**9.3** [9.0-10.9]
Beta diversity	Sorensen dissimilarity index mean [&&]	**0.758**	**0.793**	**0.809**	**0.830**	**-**
		p>0.05				

* Mean comparison was performed with Kruskall-Wallis.

** High-risk HPV detected by the LiPA25 test: 16, 18, 31, 33, 35, 39, 45, 51, 52, 56, 58, 59. Low-risk HPV detected by the LiPA25 test: 6, 11, 34, 40, 42, 43, 44, 53, 54, 66, 68/73, 70, 74. Note: Any incidence in 68/73 is count as a one HPV type.

[&] Shannon diversity refers to exp(Shannon diversity) and Simpson diversity refers to 1/Simpson index.

[&&] Sorensen index of disimilarity Bw=(b+c)/(2*a+b+c). Comparisons were performed with permutation test for homogeneity of multivariate dispersions, based in 99 permutations.

(a, b) Different letters across groups indicate significant differences, with not overlapping of the 95% confidence interval ($CI_{95\%}$).
