# Supplementary material for: High Rate of Infection by Only Oncogenic Human Papillomavirus in Amerindians
Source: mSphere. 2018 May 2;3(3):e00176-18. doi: 10.1128/mSphere.00176-18 (PMC5932372; doi:10.1128/mSphere.00176-18)
Supplement: TABLE S4 [file sph003182535st4.pdf]

Table S4

Analysis	HPV diversity measures	Amerindian subject-based urban groups Low (n=14)	Medium (n=16)	High (n=17)	Mestizos Mestizo (n=19)	All population (N=66)
Sample coverage-based	Observed richness (Hill number ***q=0)*** [$CI_{95\%}$]	**12.3** [8.5-16.1] (a)	**13** [9.6-16.4] (a)	**17.5** [10.9-24.1] (a)	**20.9** [15.5-26.3] (a)	**22** [19.1-25.0]
	Shannon diversity[&] (Hill number ***q=1***) [$CI_{95\%}$]	**8.2** [5.9-10.5] (a)	**9** [6.7-11.3] (a)	**12.1** [7.9-16.3] (ab)	**16.3** [12.2-20.5] (b)	**13** [11.4-14.6]
	Simpson diversity[&] (Hill number ***q=2)*** [$CI_{95\%}$]	**6.1** [4.2-7.9] (a)	**6.9** [4.7-9.1] (ab)	**8.6** [5.2-12.0] (ab)	**12.8** [8.6-17.1] (b)	**9.0** [7.5-10.5]
Asymptotic estimation	Observed richness (Hill number ***q=0)*** [$CI_{95\%}$]	**14.8** [11.6-35.1] (a)	**15.9** [12.6-36.3] (a)	**20.6** [14.4-53.1] (a)	**24.0** [19.2-46.4] (a)	**23.6** [39.6-21.4]
	Shannon diversity[&] (Hill number ***q=1***) [$CI_{95\%}$]	**9.8** [7.7-13] (a)	**10.8** [8.6-14.3] (a)	**14.2** [9.8-21.0] (ab)	**19.9** [14.4-25.7] (b)	**13.8** [16-12.6]
	Simpson diversity[&] (Hill number ***q=2)*** [$CI_{95\%}$]	**6.6** [5.8-9.0] (a)	**7.7** [6.7-10.2] (a)	**9.6** [7.7-14] (ab)	**15.4** [11.7-20.6] (b)	**9.1** [10.9-8.7]

& Shannon diversity refers to exp(Shannon diversity) and Simpson diversity refers to 1/Simpson index.

(a, b) Different letters across groups indicate significant differences, with not overlapping of the 95% confidence interval (CI95%)
