# Supplementary material for: High Rate of Infection by Only Oncogenic Human Papillomavirus in Amerindians
Source: mSphere. 2018 May 2;3(3):e00176-18. doi: 10.1128/mSphere.00176-18 (PMC5932372; doi:10.1128/mSphere.00176-18)
Supplement: FIG S3 [file sph003182535sf3.pdf]

HPV risk type	HPV types	HPV types frequency (%) (among infected women)									
		Community-based groups (N=83)					Subject-based groups (N=66)				
		Amerindians (N=59)				Mestizos	Amerindians (N=47)				Mestizos
		**Low (N=16)**	**Medium (N=21)**	**High (N=22)**	**All Amerindians**	**Mestizo (N=24)**	**Low (N=14)**	**Medium (N=16)**	**High (N=17)**	**All Amerindians**	**Mestizo (N=19)**
High-risk HPV	HPV18	56.3	47.6	40.9	44.6	37.5	50.0	56.3	52.9	34.8	36.8
	HPV39	50.0	47.6	31.8	37.3	25.0	64.3	43.8	41.2	31.8	26.3
	HPV16	18.8	28.6	9.1	16.9	12.5	28.6	31.3	29.4	16.7	10.5
	HPV52	18.8	9.5	27.3	18.1	16.7	7.1	12.5	11.8	10.6	21.1
	HPV59	18.8	4.8	0.0	7.2	8.3	7.1	6.3	5.9	4.5	5.3
	HPV56	6.3	19.0	13.6	12.0	8.3	14.3	18.8	17.6	10.6	5.3
	HPV31	6.3	4.8	13.6	7.2	4.2	7.1	12.5	11.8	6.1	5.3
	HPV33	0.0	9.5	0.0	3.6	4.2	0.0	0.0	0.0	0.0	5.3
	HPV51	0.0	9.5	0.0	3.6	4.2	0.0	6.3	5.9	3.0	5.3
	HPV58	0.0	4.8	0.0	4.8	12.5	0.0	0.0	0.0	0.0	15.8
	HPV45	0.0	0.0	0.0	0.0	8.3	0.0	0.0	0.0	0.0	5.3
Low-risk HPV	HPV54	12.5	4.8	0.0	7.2	12.5	7.1	6.3	5.9	3.0	15.8
	HPV66	6.3	9.5	0.0	7.2	12.5	14.3	0.0	0.0	3.0	10.5
	HPV44	0.0	14.3	4.5	9.6	16.7	0.0	12.5	11.8	3.0	10.5
	HPV68-73	0.0	9.5	4.5	6.0	8.3	0.0	6.3	5.9	4.5	10.5
	HPV43	0.0	4.8	0.0	2.4	4.2	0.0	0.0	0.0	1.5	5.3
	HPV34	0.0	4.8	0.0	1.2	0.0	0.0	0.0	0.0	1.5	0.0
	HPV53	0.0	0.0	13.6	4.8	4.2	14.3	6.3	5.9	4.5	0.0
	HPV11	0.0	0.0	4.5	1.2	0.0	7.1	0.0	0.0	1.5	0.0
	HPV70	0.0	0.0	4.5	1.2	0.0	0.0	0.0	0.0	0.0	0.0
	HPV74	0.0	0.0	0.0	2.4	8.3	0.0	0.0	0.0	0.0	10.5
	HPV6	0.0	0.0	0.0	1.2	4.2	0.0	0.0	0.0	0.0	5.3
