# Supplementary material for: High Rate of Infection by Only Oncogenic Human Papillomavirus in Amerindians
Source: mSphere. 2018 May 2;3(3):e00176-18. doi: 10.1128/mSphere.00176-18 (PMC5932372; doi:10.1128/mSphere.00176-18)
Supplement: TABLE S5 [file sph003182535st5.pdf]

Table S5

Analysis	HPV measures	Body sites Cervix (n=14)	Introitus (n=10)	Anal (n=7)	Oral (n=6)
**Prevalence and HPV counts**	Prevalence of any HPV type % (n/N); [$CI_{95\%}$] *	**77.8** (14/18) [52-93] (a)	**55.5** (10/18) [31-78] (b)	**38.9** (7/18) [18-64] (b)	**33.3** (6/18) [14-59] (b)
	N of high and low-risk HPV types	**15**	**6**	**4**	**7**
	N of high-risk HPV types	**9**	**2**	**2**	**3**
	N of low-risk HPV types	**6**	**4**	**2**	**4**
	Total HPV types detected	**18** (high- risk: HPV31, 16, 18, 31, 33, 39, 52, 56, 59; Low-risk: 11, 44, 53, 54, 66, 68, 68/73, 70, 74)			
**Sample size-based**	Observed richness (Hill number ***q=0)*** [$CI_{95\%}$] **	**12.8** [9.2-16.3] (b)	**3.1** [1.3-4.9] (a)	**5.3** [2.0-8.7] (a)	**3.3** [1.1-5.6] (a)
	Shannon diversity[&] (Hill number ***q=1***) [$CI_{95\%}$] **	**9.7** [6.2-13.1] (b)	**2.5** [1.2-3.8] (a)	**4.5** [2.2-6.9] (ab)	**2.7** [1.3-4] (a)
	Simpson diversity (Hill number ***q=2)*** [$CI_{95\%}$] **	**7.3** [4.6-10.1] (b)	**2.2** [0.9-3.4] (a)	**3.6** [0.8-6.4] (ab)	**2.2** [1.0-3.5] (a)
**Sample coverage-based**	Observed richness (Hill number ***q=0)*** [$CI_{95\%}$] **	**18.2** [12.0-24.4] (b)	**2.7** [1.4-4.1] (a)	**5.6** [2.2-9.1] (a)	**2.6** [1.4-3.7] (a)
	Shannon diversity[&] (Hill number ***q=1***) [$CI_{95\%}$] **	**12.2** [7.6-16.9] (b)	**2.3** [1.1-3.4] (a)	**4.9** [2.0-7.8] (ab)	**2.2** [1.1-3.3] (a)
	Simpson diversity (Hill number ***q=2)*** [$CI_{95\%}$] **	**8.3** [4.9-11.7] (b)	**2.0** [1.0-3.0] (a)	**3.7** [0.7-6.8] (ab)	**2.0** [1.0-2.9] (a)
**Asymptotic estimation**	Observed richness (Hill number ***q=0)*** [$CI_{95\%}$] **	**9.5** [16.0-62.6] (b)	**1.2** [3.0-10.7] (a)	**3.7** [4.3-24.8] (a)	**1.1** [3.0-10.2] (a)
	Shannon diversity[&] (Hill number ***q=1***) [$CI_{95\%}$] **	**3.5** [10.2-22.4] (b)	**1.0** [2.4-4.9] (a)	**2.7** [3.4-11.6] (ab)	**0.9** [2.4-4.6] (a)
	Simpson diversity (Hill number ***q=2)*** [$CI_{95\%}$] **	**2.0** [7.6-13.2] (b)	**0.8** [2.1-4.0] (a)	**2.4** [3.0-9.1] (ab)	**0.8** [2.1-3.8] (a)

* Analysis of HPV prevalence among groups were performed with log-lineal model.

**Shannon diversity refers to exp(Shannon diversity) and Simpson diversity refers to 1/Simpson index.

(a, b) Different letters across groups indicate significant differences. Diversity comparison was performed based on the not overlapping of the 95% confidence Interval ($CI_{95\%}$).
