# Supplementary material for: High Rate of Infection by Only Oncogenic Human Papillomavirus in Amerindians
Source: mSphere. 2018 May 2;3(3):e00176-18. doi: 10.1128/mSphere.00176-18 (PMC5932372; doi:10.1128/mSphere.00176-18)
Supplement: TABLE S6 [file sph003182535st6.pdf]

Table S6

Categories	Body site comparisons	Cohen's kappa	Cohen's kappa interpretation*	Sensibility**	Specificity***
**Any high-risk HPV type**	Cervix vs. introitus	0.26	fair	0.43	0.77
	Cervix vs. oral	0.26	fair	0.43	0.82
	Cervix vs. anal	-0.11	less than chance	0.45	0.43
	Anal vs. introitus	-0.27	less than chance	0.38	1.00
	Anal vs. oral	0.26	fair	0.40	0.77
	Oral vs. introitus	0.17	slight	0.40	0.77
**HPV18**	Cervix vs. introitus	0.18	slight	0.60	0.62
	Cervix vs. oral	-0.05	less than chance	0.43	0.91
	Cervix vs. anal	0.37	fair	0.20	0.85
	Anal vs. introitus	0.37	fair	0.40	0.92
	Anal vs. oral	0.05	slight	0.20	0.85
	Oral vs. introitus	0.17	slight	0.40	0.77

*Interpretation: < 0 less than chance; 0.01–0.20 slight; 0.21– 0.40 fair; 0.41–0.60 moderate; 0.61–0.80 substantial; and 0.81–0.99 almost perfect

**Measures the proportion of positives that are commonly identified in two body sites

***Measures the proportion of negatives that are commonly identified in two body sites
