# Supplementary material for: High Rate of Infection by Only Oncogenic Human Papillomavirus in Amerindians
Source: mSphere. 2018 May 2;3(3):e00176-18. doi: 10.1128/mSphere.00176-18 (PMC5932372; doi:10.1128/mSphere.00176-18)
Supplement: TABLE S7 [file sph003182535st7.pdf]

Table S7

Host ID	Subject-based groups	Community-based groups	Ethnicity*	Age	Cervical abnormalities type **	HPV High-risk ***	HPV Low-risk [&]
10	Medium	High	Amerindian	18	LGSIL	18, 31	44, 53
13	High	High	Amerindian	41	ASGUS	18	none
15	High	High	Amerindian	41	LGSIL	16	none
124	*Missing*[&&&]	High	Amerindian	26	LGSIL	52	none
2	Medium	High	Amerindian	35	LGSIL[&&]	31	none
70	Low	Medium	Amerindian	35	LGSIL	16, 18, 39, 56	none
69	Medium	Medium	Amerindian	25	HGSIL	16, 18, 39	none
109	*Missing*[&&&]	Low	Amerindian	46	LGSIL	18, 39, 59	none
92	Low	Low	Amerindian	33	LGSIL	16, 18	none

* Living in Amerindian villages and/or self-identified.

**LGSIL, Low Grade Squamous Intraepithelial Lesion; HGSIL, High Grade Squamous Intraepithelial Lesion; ASGUS, Atypical Squamous Glandular Cells of Undetermined Significance

***Possible high-risk HPV detected by the LiPA25 test: 16, 18, 31, 33, 35, 39, 45, 51, 52, 56, 58, 59.

[&]Possible low-risk HPV detected by the LiPA25 test: 6, 11, 34, 40, 42, 43, 44, 53, 54, 66, 68/73, 70, 74.

[&&]No abnormality detected by cytology but by biopsy

[&&&] *Missing*. No subject-based survey could be applied.
